# Risk factors for mechanical complications of peripherally inserted central catheters in children

**DOI:** 10.1017/ice.2022.193

**Published:** 2023-06

**Authors:** David J. Greencorn, Stefan Kuhle, Lingyun Ye, Kieran J. Moore, Ketan P. Kulkarni, Joanne M. Langley

**Affiliations:** 1 Faculty of Medicine, Dalhousie University, Halifax, Nova Scotia, Canada; 2 Department of Pediatrics, Dalhousie University, Halifax, Nova Scotia, Canada; 3 Department of Obstetrics and Gynaecology, Dalhousie University, Halifax, Nova Scotia, Canada; 4 Canadian Center for Vaccinology (Dalhousie University, IWK Health and Nova Scotia Health), Halifax, Nova Scotia, Canada

## Abstract

**Objective:**

To determine risk factors for mechanical (noninfectious) complications in peripherally inserted central catheters (PICCs) in children.

**Design:**

Retrospective cohort study.

**Setting:**

Pediatric tertiary-care center in Nova Scotia, Canada.

**Patients:**

Pediatric patients with a first PICC insertion.

**Methods:**

All PICCs inserted between January 2001 until 2016 were included. Age-stratified (neonates vs non-neonates) Fine–Grey competing risk proportional hazard models were used to model the association between each putative risk factor and the time to mechanical complication or removal of the PICC for reasons not related to a mechanical complication. Models were adjusted for confounding variables identified through directed acyclic graphs.

**Results:**

Of 3,205 patients with PICCs, 706 had mechanical complications (22% or 14 events/1000 device days). For both neonates and older children, disease group, lumen count, and prior leak were all associated with mechanical complications in the adjusted proportional hazards model. Access vein and prior infection were also associated with mechanical complications for neonates, and age group was associated with mechanical complications among non-neonates.

**Conclusions:**

We have identified several risk factors for mechanical complications in patients with PICCs that will help improve best practices for PICC insertion and care.

Central venous access devices (CVADs) are commonly used in children who require intravascular access for prolonged parenteral nutrition or administration of intravenous medication.^1^ Peripherally inserted central catheters (PICCs) may be more suitable than other tunneled or nontunneled lines or implanted ports for children in some situations because they can be inserted and removed without a surgical procedure in an ambulatory setting. PICCs may also have a lower risk of serious insertion complications.^1–5^

Despite being deemed very safe, PICCs are not free of complications. A systematic review found that a PICC failure, defined as the inability to complete the intended treatment via the device, occurs at a rate of 12.4 per 1,000 device indwelling days.^6^ Failures can be caused by complications, such as central-line–associated bloodstream infection, or by mechanical complications such as catheter occlusion, infiltration, migration, fracture, or disconnection.^7,8^ Rates of such mechanical complications in PICCs at our center have been reported previously at a rate of 17.3 per 1,000 days.^9^ Although most literature has focused on infectious complications or immediate events during CVAD insertion, >80% of PICC complications that occur after insertion are mechanical in nature.^10,11^

In previous research, PICCs were the most commonly used CVAD in our setting, representing 48%–54% of inserted CVADs, and mechanical complications were common.^9,12^ In this study, we sought to identify characteristics of patients and PICC device that were associated with mechanical complications to inform efforts and interventions to reduce the failure of PICC devices.

## Methods

This retrospective cohort study was conducted at the IWK Health Centre in Halifax, Nova Scotia, a pediatric and women’s university-affiliated tertiary-care center serving the 3 Maritime provinces of Canada (population 1.9 million). Children who had a CVAD inserted between January 2001 and July 2016 at this center were followed from the time of CVAD insertion until its removal. Patients aged 0–18 years whose first CVAD insertion was a PICC were eligible for this study.

The study was approved by the institutional research ethics board (file no. 1023346).

### Data sources

A data set was created from a linkage of the IWK CVAD database with the Hospital-Acquired Infections Database of the institutional Infection Prevention and Control program. The CVAD database contains records of all central-venous line placements (insertion record) and daily reports of device use since 1996. Upon insertion of a line, the date of insertion, reason for and urgency of device type, anatomic access site, insertion complications, and clinical and demographic information of the patient are documented. Over the lifespan of each device, daily data on its condition (including mechanical complications, device removal, signs of line infection, etc) are collected at the bedside by the nursing team.^12,13^ The Hospital-Acquired Infections Database captures information on targeted infections not present or incubating on admission as part of an ongoing infection prevention and control surveillance program.^12^

### Primary outcome

The primary outcome of interest in this study was the time to PICC mechanical complication. Mechanical complication was defined as any occurrence of occlusion (eg, unable to withdraw or push fluid through the line), infiltration (eg, fluid in tissues near line tip), migration (eg, line in a site different than at insertion, including outside the vessel), fracture, or disconnection. Because the daily flow sheets were not recorded on outpatients, all out-of-hospital days were assumed to have no mechanical complication based on the assumption that patients would seek medical attention for evaluation of any significant catheter-related event, and therefore would be known to institutional care teams.

### Potential risk factors

We examined the following patient- and PICC-related potential risk factors. We examined age at insertion: 0–28 days, 29 days to <6 months, 6 months to <1 year, 1 year to <3 years, 3 years to <10 years, ≥10 years. For disease group, we examined the specialty unit where the patient received the majority of their care, which did not necessarily align with the area of the hospital where the PICC was inserted: neonatal intensive care unit (NICU), general pediatric medical unit (PMU), medical-surgical nursing unit (MSNU), and other, including pediatric intensive care unit, hematology–oncology, nephrology unit, and any other area of the hospital (because there were fewer patients in these areas). We also examined urgency of insertion (elective vs nonelective), insertion side of the body (right vs left), access vein (arm, leg, or jugular), lumen count (single vs ≥2), and prior leak (defined as seepage of liquid from the site of insertion; yes or no). Finally, we assessed the role of PICC-associated bloodstream infection (yes or no).

### Statistical analysis

**Fig. 1.**
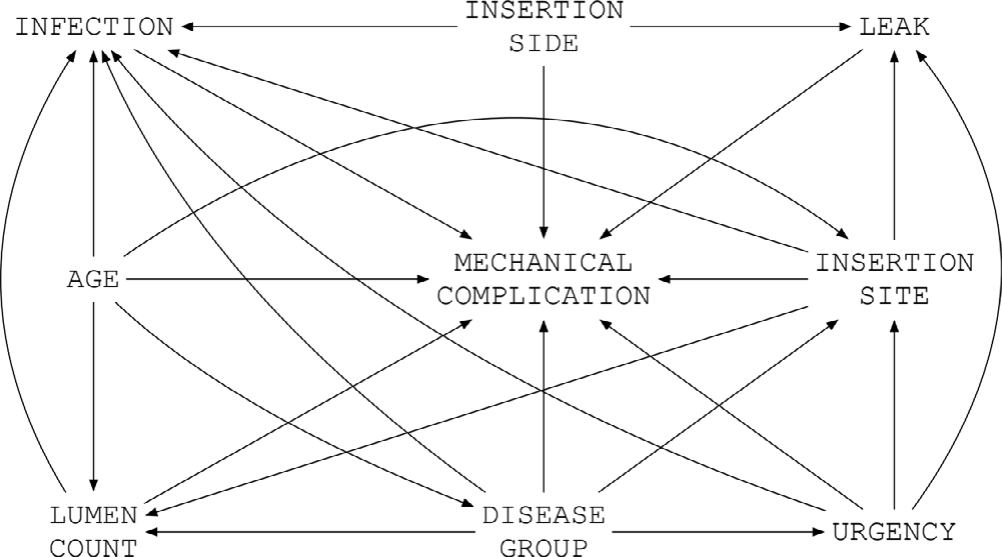
Directed Acyclic Graph for the association between patient- and catheter-related factors with mechanical complications in pediatric patients.

Event rates for mechanical complications were defined as the number of events per 1,000 device days and as the proportion of total PICCs that had a mechanical complication. Patient and PICC characteristics were summarized as relative frequencies. A Fine–Gray competing-risk proportional hazards model was used to examine the associations of PICC and patient characteristics with mechanical complications.^14^ The primary outcome was time to mechanical complication, and the competing risk was mechanical complication not related to line removal or death. Hazard ratios from the Fine–Gray model are so-called subdistribution hazard ratios and have a different interpretation than normal hazard ratios because they estimate the hazard of an event in a hypothetical population that has experienced neither the event of interest nor the competing event.^15^ Ties were handled using the Breslow approximation method. Separate models were developed for each potential risk factor^16^ with covariate adjustment sets for each model identified from a directed acyclic graph (DAG) (Fig. 1).^17,18^ Introductions to DAGs are available elsewhere.^18^ In brief, a DAG is a graphical representation of hypothesized relationships between variables relevant to the association of interest based on content expertise. Using a set of probability-theory–based rules, a DAG allows for identification of sets of confounding variables that should be controlled for to obtain an unbiased estimate of the total effect of the exposure on the outcome. Leak and infection were used as time-varying covariates in the models, and the remaining covariates were treated as time invariant. Models were additionally adjusted for period (before or after 2011) because the PICC insertion protocol changed to having a dedicated insertion team in 2011, which led to <2% of PICCs insertions occurring in the operating room thereafter. The analysis was stratified by age group: neonates (aged ≤28 days) and non-neonates (aged >28 days). We used SAS version 9.4 software (SAS Institute, Cary, NC) to perform the statistical analysis.

## Results

In total, 5,743 pediatric patients had a CVAD inserted between January 2001 and July 2016, with a total indwelling time of 780,488 days.^9^ Of these, PICCs were inserted in 3,205 (56%) patients and accumulated 50,269 (6.4%) days of indwelling time. Sample characteristics are shown in Table 1. Also, 64% of patients were neonates. Most PICC insertions were elective and were inserted into a vein of the upper extremity, with no clear preference for either side. The vast majority of the PICCs were single-lumen devices.

**Table 1.** Patient and Line Characteristics for Pediatric Patients With A Peripherally Inserted Central Catheter Implanted at the IWK Health Centre (Halifax, NS, Canada) Between 2001 and 2016 (n = 3,205)

	Nonmechanical Complications	Mechanical Complications	Overall	Missingness
Variable	No. (%)	No. (%)	No. (%)	%
**Age**				0
0–28 d	1,634 (80)	416 (20)	2,050 (64)	
29 d to < 6 mo	112 (75)	38 (25)	150 (4.7)	
6 mo to <1 y	41 (66)	21 (34)	62 (1.9)	
1 y to <3 y	92 (63)	54 (37)	146 (4.6)	
3 y to <10 y	221 (79)	60 (21)	281 (8.8)	
≥10 y	399 (77)	117 (23)	516 (16)	
**Disease group**				0
NICU	1,601 (80)	390 (20)	1,991 (62)	
PMU	418 (77)	127 (23)	545 (17)	
MSNU	339 (75)	114 (25)	453 (14)	
Other	141 (65)	75 (35)	216 (6.7)	
**Urgent insertion**				9.7
Elective	1,787 (77)	521 (23)	2,308 (80)	
Nonelective	478 (82)	107 (18)	585 (20)	
**Side of insertion**				0.031
Right	1,347 (79)	369 (22)	1,716 (54)	
Left	1,152 (77)	336 (23)	1,488 (46)	
**Catheter access vein**				1.4
Arm	1,764 (76)	547 (24)	2,311 (73)	
Jugular	282 (75)	92 (25)	374 (12)	
Leg	418 (88)	58 (12)	476 (15)	
**Lumen count**				0
1 Lumen	2,416 (79)	660 (21)	3,076 (96)	
2+ Lumen	83 (64)	46 (36)	129 (4.0)	

Note. MC, mechanical complication; MSNU, medical-surgical nursing unit; NICU, neonatal intensive care unit; PMU pediatric medical unit.

Mechanical complications occurred in 706 of the 3,205 patients (22%, or 14.0 events per 1,000 device days). The incidence of mechanical complication decreased over the study period from 14.9 per 1,000 device days (2001–2010) to 12.5 per 1,000 device days (2011–2016). The median dwell times were 8 days (range: 1–116) and 12 days (range: 1–267) for patients with mechanical complications and patients who experienced a competing event, respectively. Table 2 shows a breakdown of the types of mechanical complication.

**Table 2.** Types of Mechanical Complications Occurring in 706 of 3,205 Patients With Their First Peripherally Inserted Central Catheter^a^

Type of Mechanical Complication	No. (%)
Occlusion	358 (11)
Infiltration	199 (6.2)
Disconnect	83 (2.6)
Migration	49 (1.5)
Fracture	35 (1.1)

a Proportions are relative to the full sample; some patients had more than one mechanical complication simultaneously.

Results from the regression model are shown in Table 3. Among neonates, disease group (other vs NICU), multiple lumina, prior infection, and leak had a higher hazard of mechanical complication, whereas leg-vein PICCs had a lower hazard of mechanical complication than arm-vein PICCs. Among non-neonates, age group (1 year to <3 years vs 29 days to <6 months), disease group (PMU, MSNU, or other vs NICU), multiple lumina, and leak all had higher hazards of mechanical complication.

**Table 3.** Unadjusted and Adjusted^a^ Hazard Ratios for the Association of Patient- and Catheter-Related Factors with Mechanical Complications in Pediatric Patients With PICCs (n = 3,205), Stratified By Age Group^b^

	Neonates		Non-neonates	
	Unadjusted	Adjusted	Unadjusted	Adjusted
Variable	HR (95% CI)	HR (95% CI)	HR (95% CI)	HR (95% CI)
**Age group**				
29 d to <6 mo	…	…	Ref.	Ref.
6 mo to <1 y	…	…	1.45 (0.84–2.49)	1.36 (0.79–2.35)
1 y to <3 y	…	…	1.62 (1.07–2.47)	1.64 (1.08–2.49)
3 y to <10 y	…	…	0.82 (0.54–1.22)	0.83 (0.55–1.24)
≥10 y	…	…	0.86 (0.60–1.24)	0.85 (0.59–1.22)
**Disease group**				
NICU	Ref.	Ref.	Ref.	Ref.
PMU	1.07 (0.57–2.01)	1.10 (0.58–2.08)	3.37 (1.05–10.8)	4.75 (1.44–15.7)
MSNU	1.41 (0.76–2.62)	1.50 (0.81–2.77)	3.58 (1.11–11.6)	4.75 (1.44–15.7)
Other	2.20 (1.26–3.85)	2.28 (1.28–4.04)	4.92 (1.51–16.0)	6.87 (2.05–23.0)
**Urgency**				
Elective	Ref.	Ref.	Ref.	Ref.
Nonelective	0.78 (0.60–1.01)	0.79 (0.61–1.02)	0.82 (0.58–1.15)	0.90 (0.63–1.29)
**Insertion side**				
Right	Ref.	Ref.	Ref.	Ref.
Left	1.03 (0.85–1.25)	1.03 (0.84–1.27)	1.08 (0.86–1.36)	1.09 (0.85–1.40)
**Access vein**				
Arm	Ref.	Ref.	Ref.	Ref.
Jugular	0.88 (0.66–1.18)	0.82 (0.59–1.12)	1.34 (0.96–1.85)	0.98 (0.60–1.60)
Leg	0.49 (0.36–0.65)	0.48 (0.35–0.65)	0.55 (0.23–1.31)	0.41 (0.13–1.27)
**Lumen count**				
Single	Ref.	Ref.	Ref.	Ref.
Multiple	1.69 (1.16–2.48)	1.83 (1.10–3.06)	1.97 (1.24–3.11)	2.63 (1.44–4.82)
**Prior infection**				
No	Ref.	Ref.	Ref.	Ref.
Yes	2.06 (1.31–3.25)	2.14 (1.34–3.42)	1.65 (0.18–14.8)	1.35 (0.25–7.37)
**Prior Leak**				
No	Ref.	Ref.	Ref.	Ref.
Yes	2.11 (1.17–3.80)	2.42 (1.30–4.49)	9.76 (4.47–21.3)	16.0 (7.97–32.3)

Note. CI, confidence interval; HR, hazard ratio; MSNU, medical-surgical nursing unit; NICU, neonatal intensive care unit; PICC, peripherally inserted central catheter; PMU, pediatric medical unit; Ref, referent.

a Models were adjusted for (1) age group model (non-neonates only): insertion side, period; (2) disease group model: age group (non-neonates only), insertion side, period; (3) urgency model: age group (non-neonates only), disease group, insertion side, lumen count; (4) insertion side model: access vein, age group (non-neonates only), disease group, urgency, lumen count, period; (5) access vein model: age group (non-neonates only), disease group, urgency, insertion side, lumen count, period; (6) lumen count model: age group (non-neonates only), disease group, urgency, insertion side, access vein, leak, period; (7) infection model: age group (non-neonates only), disease group, urgency, insertion side, access vein, lumen count, leak, period; (8) leak model: age group (non-neonates only), disease group, urgency, insertion side, access vein, lumen count, infection, period.

b Neonates (aged ≤28 d) and non-neonates (aged >28 d).

## Discussion

In this analysis of PICCs inserted over 16 years of observation, we identified characteristics that were associated with increased hazard of mechanical complications across 2 distinct age strata: neonates (aged ≤28 days) and children (aged >28 days). The mechanical complication rate in our setting is comparable to those of previous reports of pediatric populations; ∼8%–28% of PICCs have some form of mechanical complication, with rates ranging from 4.3 to 19.3 mechanical complications per 1,000 device days.^2,10,19,20^ However, direct comparisons with other studies are limited because the definitions of mechanical complications varied across studies. Several studies required a failure of the PICC line in their definition of mechanical complication, whereas our study uses a broader definition that includes several categories of line malfunction: line occlusion, infiltration, disconnection, migration or fracture. Among non-neonates between 1 and 3 years of age, the hazard for mechanical complications associated with a PICC was highest, although 2 previous studies showed a decreasing trend of mechanical complication rates with increasing age.^2,10^ Two factors likely account for this finding: First, patients in this age group are more mobile than neonates. Second, the smaller-gauge lumen catheters used in younger children (2–3 French for infants up to 5 French for children aged ≥10 years) lead to an increased frequency of occlusive complications than devices with larger lumens.^21^ This observation highlights the need for securing PICCs in this age group and for adult supervision while the child is awake.

The setting where patients received most of their care (cf, disease group) was also associated with differences in hazard ratios for mechanical complications. In neonates, there was no significant difference between patients cared for in the NICU compared to the PMU or MSNU. However, we detected a higher hazard for children cared for in other areas. This finding could be attributed to more diverse indications for the PICC insertion or could be a result of fewer patients with PICCs in wards where care teams are less experienced with PICC care. Patients in the NICU generally had a lower mechanical complication hazard than the other groups, which maybe due to decreased mobility of the patients and increased measures to secure lines in place. Although leakage of fluid from the line insertion site (“Leak” variable) had a higher hazard ratio for mechanical complication, we interpret this to be a clinical manifestation of poor site healing rather than a risk factor itself.

We did not detect a difference in the incidence of mechanical complication based on PICC insertion side. Although no evidence suggests an increase in risk of complications in pediatric populations, there seems to be little consistency in the literature on whether the insertion side influences the rate of complications in adults.^22–24^ However, some vascular-access specialists prefer inserting PICCs using an access vein on the right side of adult patients, regardless of their handedness, based on limited evidence that there is a mildly increased risk of thrombotic events when inserted on the left.^22^

Risk factors for mechanical complications in PICCs that we have identified in pediatric populations can be compared to adults. Nonelective PICC insertion has been associated with increased risk of complication for PICCs in adults.^25^ However, our sample shows that nonelective PICC insertions are not a risk factor for mechanical complications in pediatric populations. In adults, patients with obesity had an increased risk of mechanical complications compared with nonobese patients, and administration of an anticoagulant was associated with a lower risk of mechanical complications.^25^

The strengths of this study include the large sample size, the prospective data collection as part of routine clinical care, and the detailed daily records for each line filled out by trained CVAD care providers. However, several limitations should be acknowledged. The sample had large variations in disease group sizes, including few patients in hematology–oncology and pediatric intensive care, which may affect the generalizability of our findings.

Also, the CVAD database does not collect patient diagnoses, and we were unable to identify whether specific groups of conditions were associated with mechanical complications. As a result, patients who were born preterm (and potentially at a higher risk for mechanical complications) were combined with term-born infants. For outpatients, daily data on the status of the line were not available. We assumed that there were no mechanical complications in their time away from hospital because they had direct access to the care team if any concerns arose; if this were not true we may have underestimated the number of complications. Moreover, the medications that were administered via the PICC were not recorded but they could affect the risk of mechanical complication.^25^

In summary, we have identified several patient and device risk factors for mechanical complications in PICC lines in children. Mechanical complications occur commonly in children with PICC lines. These patient-safety events cause increased disease burden, including the need for new insertion. We recommend that mechanical complications associated with central lines be a routinely collected patient safety measure so that future studies can investigate risk factors for CVAD and PICC mechanical complications and identify targets for improved care.
